# The challenges of transferring chronic illness patients to adult care: reflections from pediatric and adult rheumatology at a US academic center

**DOI:** 10.1186/1546-0096-7-13

**Published:** 2009-06-08

**Authors:** Aimee O Hersh, Shirley Pang, Megan L Curran, Diana S Milojevic, Emily von Scheven

**Affiliations:** 1 Department of Pediatrics, Division of Rheumatology, University of California, San Francisco, USA; 2 Department of Medicine, Division of Rheumatology, University of California, San Francisco, USA

## Abstract

**Background:**

Little is known about the transfer of care process from pediatric to adult rheumatology for patients with chronic rheumatic disease. The purpose of this study is to examine changes in disease status, treatment and health care utilization among adolescents transferring to adult care at the University of California San Francisco (UCSF).

**Methods:**

We identified 31 eligible subjects who transferred from pediatric to adult rheumatology care at UCSF between 1995–2005. Subject demographics, disease characteristics, disease activity and health care utilization were compared between the year prior to and the year following transfer of care.

**Results:**

The mean age at the last pediatric rheumatology visit was 19.5 years (17.4–22.0). Subject diagnoses included systemic lupus erythematosus (52%), mixed connective tissue disease (16%), juvenile idiopathic arthritis (16%), antiphospholipid antibody syndrome (13%) and vasculitis (3%). Nearly 30% of subjects were hospitalized for disease treatment or management of flares in the year prior to transfer, and 58% had active disease at the time of transfer. In the post-transfer period, almost 30% of subjects had an increase in disease activity. One patient died in the post-transfer period. The median transfer time between the last pediatric and first adult rheumatology visit was 7.1 months (range 0.7–33.6 months). Missed appointments were common in the both the pre and post transfer period.

**Conclusion:**

A significant percentage of patients who transfer from pediatric to adult rheumatology care at our center are likely to have active disease at the time of transfer, and disease flares are common during the transfer period. These findings highlight the importance of a seamless transfer of care between rheumatology providers.

## Background

It is estimated that 8–10% of adolescents in the United States have a chronic health condition that will require ongoing care into adulthood. This population is expected to grow as survival for children with chronic illness improves [1,2]. As a group, the pediatric rheumatic diseases, including juvenile idiopathic arthritis (JIA), systemic lupus erythematosus (SLE) and vasculitis, are among the most common chronic diseases of childhood. Juvenile idiopathic arthritis, the most common of these conditions, affects approximately 1 per 1000 children in the United States [3,4]. Although JIA remits in some patients, several recent studies have demonstrated that 30–60% percent of JIA patients continue to have active disease into adulthood [5-8]. Packham and Hall examined the clinical and functional outcomes of 246 adult patients with childhood-onset JIA and found that 43% of subjects continued to have clinically active disease after an average disease duration of 28 years. Additionally, approximately 40% of adult subjects had 'severe disability' as a result of JIA [9,10].

Remission in childhood SLE is even less common than in JIA [11]. End-organ damage, including chronic renal disease and neurologic sequelae, is more common in childhood than adult-onset SLE and can lead to significant impairment in functional outcomes and health related quality of life [12,13]. Despite substantial improvement in short-term survival during the past several decades, a significant percentage of SLE patients continue to die prematurely. In a 2004 study, the ten-year survival for childhood SLE patients was only 86%, and a recent study by Tucker et al suggests that patients with adolescent-onset SLE have a mortality rate twice that of their adult counterparts [14,15].

As a result of the chronic nature of the rheumatic diseases, most pediatric rheumatology patients require transfer of care from pediatric to adult rheumatology providers. Studies examining the transfer of care process in pediatric rheumatology and other chronic childhood diseases suggest that significant barriers exist with this process. Using data from the National Survey of Children with Special Health Care Needs, Scal et al reported on the proportion of adolescents with arthritis who received transition counseling and found that only half of young people had had a discussion with their provider about how their health needs will change into adulthood. Other transition issues that were addressed less commonly included acquiring insurance (22.5%) and transfer of care to an adult provider (19%) [16]. In a Canadian study describing the prevalence of successful transfer of care among patients with congenital heart disease, only 47% of subjects successfully transferred care, which was defined as attendance at an adult cardiology visit after discharge from pediatric cardiology care [17]. Potential barriers to successful transfer of care include factors attributable to the patient (lack of adherence to treatment and follow-up recommendations), the physician (lack of familiarity with or time to coordinate the transition process, inadequate communication between old and new providers), and the health care system (lack of insurance, limited access to care) [18-21].

In the literature, experts have drawn a distinction between the terms "transfer" and "transition" of care. "Transfer of care" is defined as the point at which a new provider assumes the medical care of a patient, while "transition of care" is defined as an "age and developmentally appropriate process, addressing the medical, psychosocial and educational/vocational aspects of care" [22]. Limited studies suggest that the transfer of care is more likely to be successful if a formal transition program is in place to prepare the patient and to facilitate the change in care providers [23,24]. In addition, there are several recent reviews which emphasize the importance of a transition program for pediatric rheumatology patients [25-30].

In order to understand the transfer of care process for young adults with chronic illness, we examined the demographic characteristics, disease outcomes and characteristics of care of a group of young adult patients who transferred care from the pediatric to adult rheumatology clinics at the University of California San Francisco (UCSF). By studying the change of care process within a single institution, we aimed to identify obstacles to care transfer, which could inform the development of a formal program for transitioning pediatric rheumatology patients to adult care at our institution.

## Methods

### Research Design

We conducted a retrospective cohort study of patients who transferred care from the pediatric to adult rheumatology clinics at UCSF between 1995 and 2005.

### Clinical Practice

During the time period of this study, there was no formal transition program in place in the Pediatric Rheumatology clinic at UCSF. The majority of the patients in the UCSF pediatric rheumatology clinic were transferred to adult care between the ages of 18 and 21. The timing of transfer was largely determined by insurance rules which determine the age of transition from pediatric to adult subspecialty care. (ex. California Children's Services mandates transition of care at age 21, regardless of disease activity or availability of an adult provider).

### Study Population

Eligible study subjects were identified by searching the adult rheumatology clinic database to find patients with a current age less than or equal to 30 years old who had at least one scheduled adult rheumatology clinic visit between 1995 and 2005. Eligible subjects were then cross referenced with the pediatric rheumatology database to determine the subset of patients who had also previously been seen in the pediatric rheumatology clinic at UCSF.

Of the 51 subjects initially identified, 15 were excluded because of unavailable records, and five were excluded because they saw a non-UCSF adult rheumatology provider after leaving pediatric rheumatology and before re-establishing care at UCSF. The subjects who were not included in the analysis because of the lack of available records did not differ from the study population with regard to diagnosis, age, sex or ethnicity. Thirty-one subjects were included in the final analysis.

### Definition of Transfer Periods

In order to compare outcomes before and after the transfer of care, two transfer periods were defined based on the date of the last pediatric rheumatology visit and the first adult rheumatology visit. The "pre-transfer period" was defined as one year +/- two months before the last pediatric visit and the "post-transfer period" was defined as one year +/- two months after the first adult rheumatology visit. We estimated that abstracting data from these time periods would allow us to measure changes in clinically relevant outcomes before and after the transfer of care.

### Measures

Relevant demographic information and clinical outcomes from the pre and post-transfer period were abstracted from the UCSF pediatric and adult rheumatology medical records for each subject (S.P.).

#### Demographic Characteristics

Information regarding various demographic characteristics was collected including age, gender, self-declared race/ethnicity, education and insurance status. Highest educational level achieved was reported in four categories: 1) high school 2) community college/vocational school 3) four year college and 4) graduate school. Insurance was categorized as public (California Children's Services or MediCal) or private (health maintenance organization (HMO) or preferred provider organization (PPO)). The distance traveled for pediatric rheumatology visits was calculated using Mapquest, an internet-based travel tool that provides a reasonable travel route and the mileage between the subject's home zip code and UCSF.

#### Clinical Characteristics

Subject diagnosis was classified in five categories: 1) systemic lupus erythematosus 2) juvenile idiopathic arthritis (JIA) 3) mixed connective tissue disease (MCTD), 4) antiphospholipid syndrome (APLS) and 5) vasculitis, using the clinical diagnosis in the medical record. For the purpose of our analysis, APLS and vasculitis were combined and analyzed as a single diagnostic category.

The Systemic Lupus Erythematosus Disease Activity Index (SLEDAI), a well validated composite measure of disease activity in SLE, was used to measure disease activity for all SLE subjects. In our clinical experience, a SLEDAI score of 1 or 2 indicates mild disease, while a SLEDAI score of = 3 indicates moderate disease. For non-SLE subjects, a global physician assessment of disease activity was determined by reviewing the clinical reports in the medical record (S.P.) and the disease activity was classified as "none", "mild/moderate" or "severe."

A complete medication list was obtained for each subject. As a measure of disease activity, the maximum prednisone dose was compared for the last pediatric and adult rheumatology visits.

The number of hospitalizations and the reasons for hospitalization (ex. disease flare versus scheduled in-patient treatment) were determined. The number of pregnancies, abortions and deaths in the cohort was also collected.

#### Appointments

The "transfer time" was defined as the time between the date of the last pediatric rheumatology visit and the date of the first appearance at an adult rheumatology visit. The number of missed pediatric and adult rheumatology appointments in the pre and post-transfer periods were also collected.

### Statistical Analysis

Baseline demographic and disease characteristics were expressed using means, standard deviations (SD) and proportions. We compared the difference in disease characteristics pre and post transition using statistical tests (rank sum, t-test, chi squared) as appropriate. For skewed data, non-parametric tests of comparison (Wilcoxon sign rank, Fisher Exact test) were used. P-values of < 0.05 were considered statistically significant.

All statistical analyses were performed using STATA software, version 9.0 (StataCorp, College Station, TX). The study protocol was approved by the UCSF Committee on Human Research.

## Results

### Patient Characteristics

The demographic and socioeconomic characteristics of the cohort are summarized in Table 1. The subject mean age at the last pediatric rheumatology visit was 19.5 years (17.4–22.0), and 74% of the cohort was female. Of the 31 subjects, approximately half had SLE (52%). Additional diagnoses included JIA (16%), MCTD (16%), APLS (13%) and vasculitis (3%) All of the subjects had graduated from high school, and 75% of the cohort continued schooling beyond high school. Educational attainment did not vary by underlying diagnosis, disease activity, or insurance status. The distance traveled to UCSF during the pre-transfer period ranged from 3 to 292 miles (median 20 miles).

**Table 1:** Subject demographics and disease characteristics.

	Cohort (n = 31)
**Age**, mean (range), y*	19.5 (17.4–22)

**Diagnosis**, No. (%)	
Systemic Lupus Erythematosus	16 (51.7)

Mixed Connective Tissue Disease	5 (16.1)

APLS/Vasculitis	5 (16.1)

Juvenile Idiopathic Arthritis/Spondyloarthropathy	5 (16.1)

**Gender**, No. (%)	

Female	23 (74)

**Ethnicity**, No. (%)	

Caucasian	10 (32.2)

Asian	7 (22.6)

Hispanic	12 (38.7)

African American	2 (6.5)

**Highest Level of Education**, No. (%)	

High school	7 (22.6)

Community College/Vocational	13 (42)

Four year college	9 (29)

Graduate school	2 (6.2)

*Age at last pediatric rheumatology visit

APLS = antiphospholipid antibody syndrome

### Outcomes

#### Disease Activity

**Figure 1:**
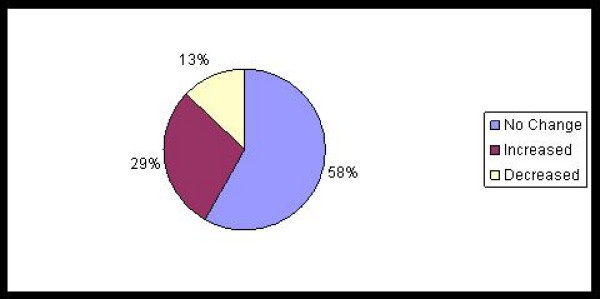
**Differences in disease activity between the Pre and Post Transfer Period (n = 31)**.

Change in disease activity pre and post-transfer was examined utilizing the SLEDAI score for subjects with SLE and the global physician assessment for non-SLE subjects. At the time of transfer, only one subject with SLE had quiescent disease (SLEDAI score = 0). Six of 16 (37%) SLE subjects had a SLEDAI score of 1–2, and 9 of 16 (56%) had a SLEDAI score = 3. In the post-transfer period, 5 subjects (31%) had experienced no change in SLEDAI score, 4 (25%) had a decrease and 7 (44%) had an increase in SLEDAI scores. There was no significant difference in the median SLEDAI scores pre and post transfer (4 (range 0–16) vs 4 (range 0–20), p = 0.3). Among non-SLE patients, 3/15 (20%) had active disease at the time of transfer; 2/15 (13%) subjects had an increase in the global physician rating of disease activity and the remaining 87% had no change in disease activity in the post-transfer assessment. These findings are summarized in Figure 1.

#### Hospitalizations

There were more hospitalizations in the year prior to transfer than the year post-transfer (32 hospitalizations among nine subjects, versus six hospitalizations among three subjects, p = 0.017). This increased rate of hospitalizations was largely due to scheduled hospitalizations for IV cyclophosphamide administration, which were more common in the pre-transfer period (21 vs 2, p = 0.03). No differences in flare-associated hospitalizations were noted between the two time periods. Hospitalizations in the pre- and post-transfer period are presented in Table 2.

**Table 2:** Changes in disease activity, hospitalizations and steroid dosing pre and post transfer.

**Disease Activity**	Pre-Transfer	Post-Transfer	P value
Median (range) SLEDAI score*	4 (0–16)	4 (0–20)	0.3

Disease activity**			

None	12 (80)	10 (67)	
Mild/moderate	2 (13)	4 (27)	0.16
Severe	1 (7)	1 (7)	

Number of Hospitalizations*			
			
Total	32	6	0.017
Scheduled	21	2	0.03
Flare associated	8	4	0.26

**Steroid Dosing**			
			
Median prednisone dose at last visit, mg (range)	5 (0–40)	2 (0–20)	0.004
Median peak prednisone dose, mg (range)	10 (0–60)	5 (0–60)	0.059
IV methylprednisolone, No (%)	3 (10)	4 (13)	0.99

* Systemic Lupus Erythematosus (n = 16)

**MCTD, APLS, Vasculitis, Spondylarthropathy (n = 15)

IV = Intravenous

#### Glucocorticoid Use

Prednisone use was common in both the pre-transfer (61%) and post-transfer (58%) periods. The mean prednisone dose was higher in the pre-transfer period than the post-transfer period (5 vs 2 mg/day, p = 0.04). Three patients received intravenous methylprednisolone during the pre-transfer period, versus four in the post-transfer period.

#### Other Medications

In the pre-transfer period, 26/31 (84%) subjects were on at least one medication for treatment of their rheumatic disease including hydroxychloroquine (61%), cyclophosphamide (13%), methotrexate (13%), azathioprine (10%), mycophenolate mofetil (13%), cyclosporine (3%), and anti-tumor necrosis factor alpha agents (13%). There were no differences in the frequency of use of these medications between the pre-transfer and the post-transfer periods.

#### Pregnancies and Abortions

In the pre-transfer period 3 of 23 female subjects became pregnant; all three pregnancies were electively terminated. In the post-transfer period there was one pregnancy which was electively terminated.

#### Death

During the post-transfer period there was one death. The patient was a 19 year old young woman with SLE who transferred care when she was 18 years old because of a change in her insurance coverage. The patient had active SLE (SLEDAI score = 4) at the time of transfer, and her SLE remained poorly controlled during the transfer period. It is noted that the patient had 3 missed appointments in the adult rheumatology clinic in the post-transfer period. The patient died from complications of SLE including diffuse mesenteric vasculitis resulting in gastrointestinal hemorrhage and sepsis.

#### Insurance

As summarized in Table 3, there was a significant change in insurance status pre and post transfer. In the pre-transfer period 75% of subjects had public insurance. In contrast, subjects were more likely to have private insurance (55% vs 45% p = 0.037) in the post-transfer period. There was no association between insurance status and educational achievement, transfer time or distance traveled to UCSF for appointments.

**Table 3:** Differences in insurance status pre and post-transfer.

**Insurance**	Pre-Transfer No. (%)	Post-Transfer No. (%)	P value
Public	23 (74)	14 (45)	0.037
	
Private	8 (26)	17 (55)	

#### Transfer time and missed clinic appointments

The median transfer time was 7.1 months (0.7 to 33). Nine of 31 subjects (29%) subjects missed their first scheduled adult rheumatology visit. No specific predictors of increased transfer time, such as distance traveled or insurance status, were identified. The number of subjects who missed scheduled appointments was high in both the pre and post transfer periods. In both time periods 10/31 (32%) subjects had at least one missed appointment. The rates of missed appointments in the pre and post transfer periods were 15% and 21% respectively.

## Discussion

As the population of children with chronic illness expands, it is essential to understand how processes of care, such as the transfer of medical care from one provider to another, affect the short and long-term outcomes of our patients. The primary goal of this study was to describe a population of pediatric rheumatology patients during the time of transfer of care, and to determine if there was a significant change in disease outcomes, such as disease activity, during this critical time period. In addition, we hoped to improve our understanding of the health care needs of young adults with chronic rheumatic disease.

In the population of patients that we examined, the majority (58%) of subjects had active disease at the time of transfer. In addition, most were taking at least one medication to treat their rheumatic disease, and over half were hospitalized in the year prior to their transfer of care. Similar findings were seen in a study of a British cohort of adolescents with JIA, in which almost 80% of 17 year olds had active disease prior to transfer from pediatric to adult rheumatology, and almost two-thirds were on a DMARD [31]. These findings highlight the importance of creating a transition system which provides seamless transfer of care between providers in order to meet the needs of these medically complicated patients.

Because of the difficulties that can arise in the transfer of care process, we hypothesized that there would be an increase in disease activity around the time of transfer. Although it is likely that our analysis was underpowered to demonstrate a statistically significant difference in median SLEDAI/global assessment scores, it is notable that 29% of subjects experienced a worsening of disease activity during the post-transfer follow-up time period. This increase in disease activity may be a result of the natural history of the disease process, but it may also reflect poor outcomes related to the transfer process. The pediatric solid organ transplant literature suggests that the transition from late adolescence to young adulthood is one of the most vulnerable periods for rejection and graft loss, due to a variety of factors, including medication non-adherence [32]. Regardless of the cause, the high rate of disease flare in the post-transfer period emphasizes the need for a comprehensive and dependable process for these patients during this critical period.

Despite evidence of high disease activity, we were surprised that the average transfer time between the last pediatric rheumatology and first adult rheumatology visit, within the same institution, was over seven months. The number of missed appointments, which is likely a proxy for patient compliance, was high in both the pre and post-transfer periods, and may have contributed to the delay between the last pediatric and first adult visit. Given the complex medical needs of this population, any future transition program at our institution will address the obstacles that lead to delays to the first adult rheumatology appointment.

With regard to insurance, a significant percentage of subjects in our study transferred from public to private insurance, and all subjects had obtained insurance coverage in the post-transfer period. A recent study by Lotstein, et al highlighted the difficulties with access to care for youth with special health care needs living near Los Angeles, California [33]. This study demonstrated that among a population of young adults with chronic disease, aged 21–24 years, 27% were without health care following discontinuation of their public insurance at the age of 21, and 39% had delays in necessary care as a result of lapses in insurance coverage. Given this data, it is likely that health outcomes could be worse for uninsured patients with chronic rheumatic disease.

Although the studies describing the clinical outcomes for pediatric rheumatology patients after transfer of care are limited, a small number of studies have examined the short term outcomes of patients who have transferred care in other health care systems or other disciplines. A recent study from France by Dugueperoux et al, examined the clinical changes of young adults with cystic fibrosis (CF) during transition from a pediatric to adult CF center [34]. In this population, disease activity remained clinically stable over the transition period. In the discussion, the authors note that given the flexibility in the timing of transfer, all of the patients transferred care at a time when they had stable disease. In the United States, timing of transfer of care is often determined by insurance age limits, and not based on the patient's optimal clinical status.

We observed some important outcomes related to transfer of care and identified interesting trends which deserve further exploration in future prospective studies. However, there are several important limitations that need to be considered. Although few studies have described the outcomes of pediatric rheumatology patients after their transfer of care, the sample size for our study was small, thereby limiting the power for some of our analyses. The retrospective nature of this study limited the ability to assess certain factors which could affect the transfer of care process including self-management abilities, developmental maturity, medication adherence and vocational and educational attainment; all essential issues which would be addressed in a comprehensive transition program. In addition, as a result of the study design, disease activity was assessed through chart evaluations, and not through direct patient examination. Because of record availability, this study only examines the outcomes of subjects transferred within our institution, which limits generalizability to other settings. In our experience, the location of adult rheumatology follow-up is determined by multiple factors including the complexity of the patients' medical condition, post-transfer insurance status and the availability of adult rheumatologists in their local communities.

## Conclusion

This study was conducted to understand the short-term outcomes of patients who transferred care from pediatric to adult rheumatology at our institution. We intend to use these findings to inform the development of a formal transition program that will prepare our patients for a successful transition to adult care. Larger, prospective longitudinal studies with longer follow-up will be necessary to better understand the health care needs of adolescents and young adults with chronic illness as they make the critical transition from adolescence into adulthood.

## Competing interests

The authors declare that they have no competing interests.

## Authors' contributions

EVS and SP were responsible for study design. SP acquired the data. EVS and AH performed the statistical analysis and AH, EVS, DM, MC interpreted the data. AH, EVS, DM and MC were responsible for the manuscript preparation. All authors read and approved the final manuscript.
